# Rumination, Posttraumatic Stress Disorder Symptoms, and Posttraumatic Growth Among Wenchuan Earthquake Adult Survivors: A Developmental Perspective

**DOI:** 10.3389/fpubh.2021.764127

**Published:** 2022-01-04

**Authors:** Wenjian Xu, Chengxiang Feng, Wanjie Tang, Yifan Yang

**Affiliations:** ^1^Department of Sociology and Psychology, School of Public Administration, Sichuan University, Chengdu, China; ^2^Institute of Psychology, Sichuan University, Chengdu, China; ^3^Center for Educational and Health Psychology, Sichuan University, Chengdu, China; ^4^School of Public Administration, Southwest Jiaotong University, Chengdu, China

**Keywords:** earthquake rumination, posttraumatic growth, perceived social support, developmental perspective, posttraumatic stress disorder symptoms

## Abstract

This study examined the long-term effects of the Wenchuan earthquake among adult survivors. Specifically, it explored the role of perceived social support (PSS) in the relationship between rumination and posttraumatic growth (PTG) and posttraumatic stress disorder (PTSD) symptoms. Data were collected from March to July 2020 using a youth survivor sample (*n* = 476) of the 2008 Wenchuan earthquake. Participants were divided into three groups depending on their age when the quake occurred: 6–11 years (*n* = 227), 12–15 years (*n* = 83), 16–19 years (*n* = 166). The results indicated that long-term PTG and PTSD symptom levels varied by age group. Both intrusive and deliberate ruminations had a significant effect on PTG as well as PTSD symptoms. PSS played a mediating role between rumination and PTG, and the mediation mechanisms varied by age group (developmental stages). Moderated analyses revealed that PSS from significant others significantly buffered the indirect effect of rumination on PTSD symptoms. Our findings demonstrated the universal nature of traumatic events encountered during childhood and adolescence development and underscore the importance of examining the developmental context of PTG in investigations on traumatic experiences and their consequences.

## Introduction

Traumatic events such as sexual and physical assault, illness, accidents, and natural disasters are common worldwide (1–5). They are a risk factor for mental health disturbance development. Posttraumatic stress disorder (PTSD) symptoms are a frequent negative psychological outcome after traumatic event exposure in both developed (4, 6–8) and developing countries (9, 10). PTSD results in extreme distress and functional impairment, and as a long-term consequence, it increases the risk of developing physical health problems (e.g., pain, heart disease, and stroke), psychological distress (e.g., depression, anxiety disorders, and suicide attempts), poor quality of life, and low life satisfaction (4, 6).

Nevertheless, a positive psychological perspective that changes the focus of trauma research to concepts such as posttraumatic growth (PTG) has been increasingly recognized, especially in the non-artificial trauma psychology field [such as earthquake and COVID-19 pandemic; (11, 12)]. Stewart (13) defined PTG as positive life changes that follow traumatic events. The concept of PTG has become more commonly used alongside posttraumatic stress to describe psychosocial functioning, especially in adults (14). However, less is known about the long-term consequences of trauma occurring during childhood and adolescence, a stage of rapid development when cognition and emotion are susceptible to trauma impact (7).

### PTG and PTSD Symptoms

Cross-sectional (4) and longitudinal (5, 15) evidence indicate that PTG and PTSD symptoms are independent constructs, which co-occur in survivors after traumatic exposure to a natural disaster. However, the relationship between PTG and PTSD symptoms remains inconclusive (4). Studies have reported a significant positive relationship (9), negative relationship (2), or no relationship at all (1, 5, 16). These inconsistent findings confound our understanding of PTSD symptoms and PTG constructs. It is necessary to clarify the relationship between PTG and PTSD symptoms. Therefore, based on this suggestion, this study aims to simultaneously examine risk and predictive mechanisms of PTG and PTSD symptoms among Wenchuan earthquake adult survivors.

### Rumination and PTG

Rumination has been described as a cognitive vulnerability typically beginning in the aftermath of a traumatic exposure that challenges the individual's framework for understanding the world (17). A distinction exists between two types of rumination about the negative stimulus, and includes intrusive and deliberate ruminations. Intrusive rumination represents thoughts that involuntarily invade one's cognitive world and generally involve a negative focus on the trauma, whereas deliberate rumination involves the deliberate reexamining of and contemplation about the experience (1, 15). The impact of intrusive and deliberate ruminations on PTG is demonstrated in cross-sectional studies (18, 19) and longitudinal research (1, 15, 20–22) on natural disasters, but some contradictions remain. For examples, deliberate rumination, not intrusive rumination was the only significant factor positively predicting PTG in children survived Hurricane Katrina (20) and adolescents after earthquake (1, 15, 21) or after a tornado (19). Deliberate rumination contributes individuals to thinking the positive aspects about traumatic events, which helps them actively construct their understanding of the post- traumatic world and attribute meaning to the trauma, thus promotes PTG (1, 21). However, Kilmer et al. (22) re-analyzed the traumatized children after Hurricane Katrina nearly 2 years after the disaster, finding baseline intrusive rumination as the only positive and significant predictor of PTG in the same sample. Therefore, further research, especially on the long-term effect on PTG is needed to clarify discrepancies.

### Perceived Social Support and PTG

The availability of PSS can facilitate the process of change by initiating communication about traumatic experiences (23, 24), leading individuals to reframe and reconstruct worldviews after a traumatic event, and creating the potential for PTG. The main-effect hypothesis of social support suggests that social support plays an independent role in reducing stress (25), and is a protective factor for PTG (11, 26). Tedeschi and Calhoun's (24) model regarding cognitive processing of PTG suggests that social support provides trauma-exposure survivors a safe environment where they can talk freely to others about their experiences, associated perceptions, and emotions. Wang et al. suggest that social support encourages survivors to establish good relationships, and promotes PTG (16). Therefore, PSS seems to lead survivors to reframe traumatic events and reconstruct their worldviews, and thus could be an important factor in predicting PTG.

### Rumination and PTG: Mediating Role of PSS

Both deliberate rumination and PSS, in addition to the reappraisal of core beliefs, reflect higher levels of PTG (18, 21). Direct correlations between deliberate rumination and PTG were found in numerous studies across various cultures (1, 27). Deliberate rumination could help individuals seek and receive more PSS to understand traumatizing events. Specifically, deliberate rumination can motivate trauma survivors to enhance their cognition regarding traumatic events, and this process encourages them to rethink their view of the self, others, and the world, and thus can be beneficial in their perception of social support (1, 19). For example, Wang et al. found that deliberate rumination contributed to PTG in adolescent trauma survivors after the Ya'an earthquake in China (1). Additionally, PSS can lessen the negative impact of intrusive rumination on individuals and increase positive experiences, leading to PTG (21). Therefore, PSS may be a mediator in the relationship between deliberate rumination and PTG.

### Rumination and PTSD Symptoms

Rumination in PTSD symptoms may reflect a deliberate attempt to understand the traumatic event and “work through” it, albeit unproductively. Over time, this may become a more automatic, default response style (28). Individuals who ruminate often report feeling they are adaptively coping by finding reasons for their distress (29). Furthermore, rumination has been shown to prospectively predict PTSD symptoms (30). The cognitive PTSD model of PTG (31) suggests that the predominant emotions experienced by trauma survivors provide an invaluable clue to cognitive themes; feelings such as fear and sadness can lead survivors to engage in maladaptive cognitive processing styles such as rumination. Ruminative thinking maintains symptoms through both cognitive and emotional processes. Specifically, individuals may ruminate to avoid traumatic memories and associated negative emotions, which temporarily alleviates distress, but ultimately interferes with the process of adaptive recovery (32).

### PSS and PTSD Symptoms

PSS is also a preventive factor for PTSD development, especially in trauma-affected communities (10). According to the buffering effect model, survivors with strong PSS can reduce the adverse psychological response caused by stress (25). Specifically, the incidence of post-traumatic stress increased in those with low PSS. PSS can provide necessary resources for adolescents surviving an earthquake to cope with negative mental reactions (4), thereby promoting coping self-efficacy. Therefore, PSS can buffer the effects of traumatic experiences on PTSD symptoms.

### Rumination and PTSD Symptoms: Moderating Role of PSS

PSS interacts with intrusive rumination and the perceptions of entrapment, thereby resisting PTSD symptom development (33). As Lepore (34) outlines in his social-cognitive theory, social support is crucial in perpetuating or breaking the pathway from trauma experiences to PTSD symptoms via rumination. Therefore, although rumination increases attention on negative aspects of a traumatic experience, the derived negative emotions should be ameliorated by others' understanding. Thus, PTSD symptoms will ease. By contrast, it is hard for people to process and regulate rumination-elicited negative emotions when they lack support (35). Thus, we propose that PSS might moderate the function of rumination on PTSD symptoms.

### Developmental Perspective on PTG and PTSD Symptoms

Age has been found to contribute to the development of psychiatric disorders (36), and both linear and curvilinear relationships have been reported between age and PTSD symptom development risk following a vehicle accident (37). The developmental periods of childhood, adolescence, young adulthood, and older adulthood are marked by age-related changes in cognitive, emotional, and social processes that may influence the likelihood of negative psychological outcomes following trauma exposure, beyond the role of sociodemographic factors (7). In a study of the COVID-19 pandemic, adolescents were significantly more likely to report clinical PTSD symptoms than adults (38). A comparative study from Saõ Paulo involving young people (aged 15–24 years) and adults reported that exposure to traumatic events was higher in the young compared with adults (39). The specific vulnerability of children and adolescents to traumatic events happens because they are in a critical developmental stage when the brain is still maturing; when the physical and psychological and personal strengths are not crystallized, and robust individual coping strategies and socioeconomic stabilities have not been fully attained (40). A rising trend with age is often found, particularly during the drastic transition period from childhood to adolescence. In a sample of children aged 10–16 years old exposed to war experiences in Lebanon, older children had more severe anxiety and depression symptoms than younger children (26). Longitudinal investigation demonstrated that adolescents at the age of 12 or 13 years suffer more frequent and serious psychological distress than their younger counterparts aged between 9 and 11 years (41, 42). Similarly, McDermott and Palmer (43) found children of grades 7–9 reported the most emotional distress compared with younger children (grades 4–6) and older adolescence (grades 10–12), following a bushfire disaster. Added to this, childhood and adolescence trauma-exposed depressive symptoms may last many years or even increase in adulthood (44, 45). However, little is known about developmental differences in response to post-disaster trauma especially regarding earthquakes. Based on previous studies, and from a developmental perspective, we assumed that the impact of the 2008 Wenchuan earthquake on adolescents would be more severe than other younger survivors.

### Aims and Hypotheses

The purpose of our study is to clarify the relationship between rumination and PTG, studying the long-term effects of trauma during childhood and adolescence using a youth survivor sample of people who experienced the 2008 Wenchuan earthquake, and to explore the mediating and moderating role of PSS in the relationship between rumination and PTG and PTSD symptoms, respectively. The current study expands the literature by providing counseling implications for prevention and intervention practice in the field of trauma-exposed psychopathology.

Specifically, we hypothesized that: 1) Long-term affected PTG and PTSD levels would be significantly different among different age groups of children who lived through the same earthquake; 2) There would be a significant correlation between intrusive rumination and deliberate rumination; both would have a significant effect on PTG; 3) PSS would mediate the link between rumination and PTG, and the mediation mechanisms would vary by age group; 4) Both intrusive and deliberate ruminations would have a significant effect on PTSD symptoms; 5) PSS would be a significant moderator for the path between rumination and PTSD symptoms.

## Methods

### Participants and Procedure

The study was conducted in Dujiangyan city in Sichuan province, which was most severely affected by the 2008 Wenchuan Earthquake in China (46). Convenience sampling offline and online approach was used to recruit young adults from March to July 2020. Psychological master-student volunteers living locally conducted participants invitation from communities and supermarkets as well as social network resources from families/relatives and friends. Additionally, volunteers also invited participants using social networking platforms such as Sina Weibo, Wechat, and QQ groups related to Dujiangyan city themes. Informed consent was obtained from all participants by their reading the description and purpose of the study and clicking “continue” to complete a questionnaire available at the professional survey Web site Wenjuanxing. Out of 560 replies, 84 were discarded from the analysis due to the incomplete psychometric instruments or excluded if they: 1) were not in Dujiangyan when the earthquake occurred; 2) were younger than 18 or older than 31. The final sample was comprised of 476 youth survivors with a mean age of 24.69 years (*SD* = 4.25), 44.54% of the sample were female (*n* = 212). Survivors were each valid participant completed written informed consent forms and was provided with 15 RMB as compensation. This study was approved by the Ethics Committee of the West China Medical Center of Sichuan University.

### Measures

#### PTG

PTG was measured using the Posttraumatic Growth Inventory (24) of the Chinese version (47). This 21-item scale consists of five subscales: 4-item personal strength, 5-item new possibilities, 7-item relating to others, 3-item appreciation of life, and 2-item spiritual change. Each item was scored on a 6-point scale (ranging from 0 = no change to 5 = very great change) and higher scores indicated better PTG. The PTG inventory has good internal consistency and good construct, convergent, and discriminate validities (24). The scale was translated into Chinese and then back-translated by three English professionals. This instrument had adequate internal reliability (α = 0.96).

#### PTSD Symptoms

PTSD symptoms over the previous month were assessed using the PTSD Checklist-Civilian Version (48). This 17-item scale has three dimensions: 5-item intrusion (e.g., “Suddenly acting or feeling as if a stressful experience were happening again”), 7-item avoidance (e.g., “Avoid thinking about or talking about a stressful experience from the past or avoid having feelings related to it”), and 5-item hyper-arousal (e.g., “Feeling jumpy or easily startled”). Each item was scored on a 5-point scale (ranging from 1 = not at all to 5 = extremely) and higher scores indicated more severe psychological distress symptoms. Probable PTSD Symptoms was classified when individual's score ≥50 (49). The Chinese version of the scale was translated by Shi and colleagues (50). Cronbach's alpha of this instrument was 0.95.

#### Rumination Inventory

Rumination was measured using the revised Event-Related Rumination Inventory (51, 52). This 20-item scale included two dimensions: 10-item intrusive rumination (such as “I cannot help thinking about the Wenchuan earthquake”) and 10-item deliberate rumination (such as “I thought about what I could learn from the Wenchuan earthquake experience”). Each item was scored on a 4-point scale (0 = not at all to 3 = always). The Chinese version of the scale was translated by Wu and colleagues (15, 52) and examined in Chinese adolescent samples, the fit indices from a confirmatory factor analysis were good and the internal reliability assessment yielded Cronbach's α of 0.88 and 0.89 for intrusive and deliberate ruminations, respectively. In this study, Cronbach's alpha for intrusive and deliberate ruminations were 0.96 and 0.96, respectively.

#### PSS

PSS was measured using the revised Perceived Social Support Scale (53). This 20-item scale consists of three subscales relating to the source of the support: 8-item regarding family (such as “My family really tries to help me”), 8-item regarding friends (such as “I can count on my friends when things go wrong”), and 4-item regarding a significant other (such as “There is a special person in my life who cares about my feelings”). Each item was scored on a 7-point scale (1 = very strongly disagree to 7 = very strongly agree) and higher scores indicated more PSS. The Chinese version of the scale was translated by Huang and colleagues, and it had adequate internal reliability and validity (54). Cronbach's alpha for family, friends, and a significant other of PSS subscales were 0.95, 0.94, and 0.92, respectively.

### Data Analysis

Frequency and descriptive analyses were conducted. Independent-sample *T*-test was performed to compare the gender characteristic. One-way ANOVA was used to examine the effects of age at the commencement of rumination, PSS, PTG, and PTSD symptoms. Pearson's and Spearman's rho correlations were computed to examine the relationships between variables. Analyses were conducted using SPSS (version 22.0). Structural equation modeling (SEM) with maximum likelihood estimation was employed to explore the hypothesized mediation model using AMOS (version 22.0). Indices used to evaluate the model fit were the Comparative Fit Index (CFI), Normed Fit Index (NFI), Incremental Fit Index (IFI), and the Root Mean Square Error of Approximation (RMSEA). Linear regression analyses were used to examine the moderation roles of PSS between intrusive and deliberate ruminations and PTSD symptoms.

## Results

### Preliminary Analyses

In the overall sample, the mean score of PTG was 89.51 (*SD* = 23.00); the prevalence of PTSD symptoms was 16.18% (scores ≥ 50, *n* = 77). Participants were divided into three groups depending on their age of developmental characteristics (41) when the Wenchuan Earthquake occurred: 6–11 years (childhood, *n* = 227), 12–15 years (early adolescence, *n* = 83), 16–19 years (late adolescence, *n* = 166). Means and *SD* of the variables are shown in Table 1. As age increased, PTG scores increased from ages 6–15 years (although age 11 was the lowest, PTG mean = 81.97), and then it decreased for ages 15–19 years old. One-way ANOVA results showed significant differences among the three age groups: intrusive rumination (*F* = 6.81, *p* = 0.001), deliberate rumination (*F* = 9.97, *p* < 0.001), PTG (*F* = 5.99, *p* < 0.01), PTSD symptoms (*F* = 3.96, *p* < 0.05). *Post-hoc* pairwise comparisons indicated that based on the degree of scores, the three age groups ranked in the following order: early adolescence, and childhood equals late adolescence on intrusive rumination, deliberate rumination, PTG, and PTSD symptoms. Men were significantly higher than women on PTG (*t* = 3.31, Cohen's *d* = 0.30, *p* < 0.01), while there were no significant differences by gender on PTSD symptoms.

**Table 1:** Means, standard deviations, and differences of variables of 2008 Wenchuan earthquake youth survivors by age (*n* = 476).

	**Age 6–11 (227)**	**Age 12–15 (83)**	**Age 16–19 (166)**	***F***	***p***	***Post-hoc* comparisons^a^**
		**M** **±*****SD***				
Intrusive rumination	16.61 (6.78)	10.76 (7.42)	16.78 (6.85)	6.81	0.001	2 > 1 = 3
Deliberate rumination	18.94 (7.34)	22.88 (7.01)	19.67 (7.34)	9.97	< 0.001	2 > 1 = 3
PSS from family	43.57 (10.23)	46.19 (9.35)	45.99 (8.28)	4.15	< 0.001	2 = 3 > 1
PSS from Friends	44.38 (7.93)	45.01 (7.15)	43.63 (8.58)	0.89	0.410	–
PSSSO	20.58 (5.27)	21.98 (4.66)	19.60 (5.98)	5.36	0.005	2 > 1 = 3
PSS	108.53 (20.23)	113.18 (18.80)	109.58 (19.93)	1.70	0.183	–
PTG	87.22 (22.98)	97.22 (19.71)	88.80 (23.85)	5.99	0.003	2 > 3 = 1
PTSD symptoms	33.48 (14.47)	38.27 (14.35)	32.89 (15.87)	3.96	0.020	2 > 1 = 3

*PSS, perceived social support; PSSSO, PSS from significant others; PTG, posttraumatic growth; PTSD, posttraumatic stress disorder*.

a *p at 0.05 level*.

### Correlation Analyses

Correlations are shown in Table 2. Significant correlations exit between PTG and PTSD symptoms (*r* = 0.30, *p* < 0.01) but partial correlation analysis (intrusive and deliberate ruminations, and PSS as covariates) were not related between PTG and PTSD symptoms (*r* = 0.07, *p* > 0.05).

**Table 2:** Correlations of variables of Wenchuan earthquake youth survivors.

**All participants**	**1**	**2**	**3**	**4**	**5**	**6**	**7**	**8**	**9**	**10**
1. Age	1	-	-	-	-	-	-	-	-	-
2. Sex	−0.08	1	-	-	-	-	-	-	-	-
3. Intrusive rumination	0.03	−0.11*	1	-	-	-	-	-	-	-
4. Deliberate rumination	0.05	−0.18^**^	0.83^**^	1	-	-	-	-	-	-
5. PSS from family	0.14^**^	−0.04	0.18^**^	0.24^**^	1	-	-	-	-	-
6. PSS from friends	0.01	0.00	0.14^**^	0.21^**^	0.69^**^	1	-	-	-	-
7. PSSSO	−0.01	−0.05	0.22^**^	0.26^**^	0.51^**^	0.60^**^	1	-	-	-
8. PSS	0.06	−0.04	0.20^**^	0.27^**^	0.90^**^	0.90^**^	0.76^**^	1	-	-
9. PTG	0.04	−0.16^**^	0.40^**^	0.46^**^	0.38^**^	0.34^**^	0.34^**^	0.41^**^	1	
10. PTSD symptoms	−0.04	−0.01	0.74^**^	0.60^**^	0.04	0.01	0.12^**^	0.06	0.30^**^	1
**Age 6–15 (*****n*** **=** **310)**										
1. Age	1	-	-	-	-	-	-	-	-	-
2. Sex	−0.15^**^	1	-	-	-	-	-	-	-	-
3. Intrusive rumination	0.18^**^	−0.11^*^	1	-	-	-	-	-	-	-
4. Deliberate rumination	0.26^**^	−0.16^**^	0.84^**^	1	-	-	-	-	-	-
5. PSS from family	0.22^**^	−0.09	0.19^**^	0.25^**^	1	-	-	-	-	-
6. PSS from friends	0.10	−0.04	0.13^*^	0.22^**^	0.71^**^	1	-	-	-	-
7. PSSSO	0.17^**^	−0.05	0.19^**^	0.26^**^	0.56^**^	0.53^**^	1	-	-	-
8. PSS	0.19^**^	−0.07	0.19^**^	0.28^**^	0.93^**^	0.88^**^	0.74^**^	1	-	-
9. PTG	0.19^**^	−0.15^**^	0.37^**^	0.42^**^	0.40^**^	0.38^**^	0.30^**^	0.42^**^	1	-
10. PTSD symptoms	0.13^*^	−0.04	0.71^**^	0.59^**^	0.02	0.01	0.11	0.04	0.27^**^	1
**Age 15–19 (*****n*** **=** **187)**										
1. Age	1	-	-	-	-	-	-	-	-	-
2. Sex	0.06	1	-	-	-	-	-	-	-	-
3. Intrusive rumination	−0.18^*^	−0.13	1	-	-	-	-	-	-	-
4. Deliberate rumination	−0.26^**^	−0.22^**^	0.82^**^	1	-	-	-	-	-	-
5. PSS from family	−0.11	0.09	0.20^**^	0.27^**^	1	-	-	-	-	-
6. PSS from friends	−0.02	0.06	0.15^*^	0.19^**^	0.70^**^	1	-	-	-	-
7. PSSSO	−0.11	−0.02	0.25^**^	0.26^**^	0.49^**^	0.72^**^	1	-	-	-
8. PSS	−0.08	0.04	0.22^**^	0.27^**^	0.86^**^	0.93^**^	0.81^**^	1	-	-
9. PTG	−0.24^**^	−0.14^*^	0.45^**^	0.54^**^	0.38^**^	0.32^**^	0.43^**^	0.42^**^	1	-
10. PTSD symptoms	−0.10	0.02	0.76^**^	0.60^**^	0.11	0.06	0.17^*^	0.12	0.36^**^	1

*PSS, perceived social support; PSSSO, PSS from significant others; PTG, posttraumatic growth; PTSD, posttraumatic stress disorder*.

* *p < 0.05*.

** *p < 0.01*.

Deliberate rumination was most significantly associated with PTG (*r* = 0.46, *p* < 0.01), followed by PSS (*r* = 0.41, *p* < 0.01), intrusive rumination (*r* = 0.40, *p* < 0.01), and gender (*r* = −0.16, *p* < 0.01). Age was not significantly related to PTG in all participants but positively correlated in the childhood group (*r* = 0.19, *p* < 0.01), while it was negatively correlated in the late adolescence group (*r* = −0.24, *p* < 0.01). Intrusive rumination was most significantly associated with PTSD symptoms (*r* = 0.74, *p* < 0.01), followed by deliberate rumination (*r* = 0.60, *p* < 0.01) and PSS from significant others (PSSSO, *r* = 0.12, *p* < 0.01).

### Mediation Analyses of PTG

**Figure 1:**
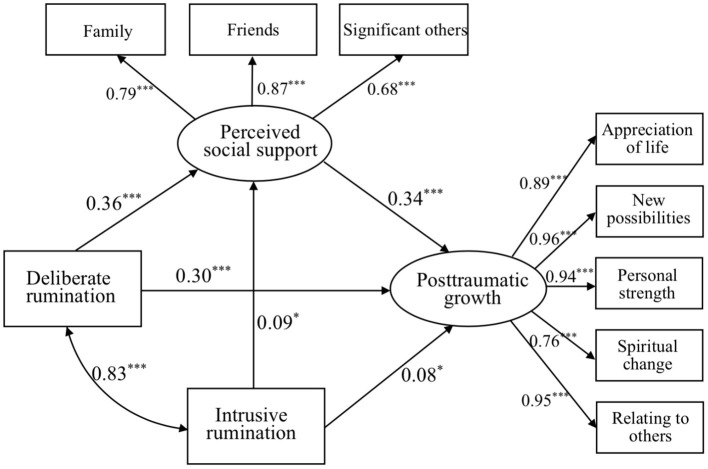
Mediation model of deliberate and intrusive ruminations to PSS and to PTG. *p < 0.05, **p < 0.01, and ***p < 0.001.

Mediation effects of PSS on the associations between intrusive rumination, deliberate rumination, and PTG of mediation model 1 are shown in Figure 1 and Table 3. The SEM model revealed that deliberate rumination (*B* = 0.30) and PSS (*B* = 0.34) had significant direct effects on PTG; intrusive rumination was related to deliberate rumination. Intrusive rumination had an indirect effect on PTG via PSS: Intrusive rumination → PSS → PTG (*B* = −0.03). Deliberate rumination had an indirect effect on PTG via PSS: Deliberate rumination → PSS → PTG (*B* = 0.12). Model 1 showed an acceptable fit for the data (χ^2^*/df* = 2.62, RMSEA = 0.058, CFI = 0.99, NFI = 0.98).

**Table 3:** Fit statistics for mediation models.

**Mediation model**	***χ^2^***	***df***	***χ^2^/df***	**GFI**	**NFI**	**IFI**	**TLI**	**CFI**	**RMSEA**	***p***
1	81.86	31	2.62	0.97	0.98	0.99	0.98	0.99	0.058	<0.001
2a	70.05	38	1.84	0.96	0.98	0.99	0.98	0.99	0.052	0.001
2b	92.36	38	2.43	0.92	0.95	0.97	0.95	0.97	0.088	<0.001

**Figure 2:**
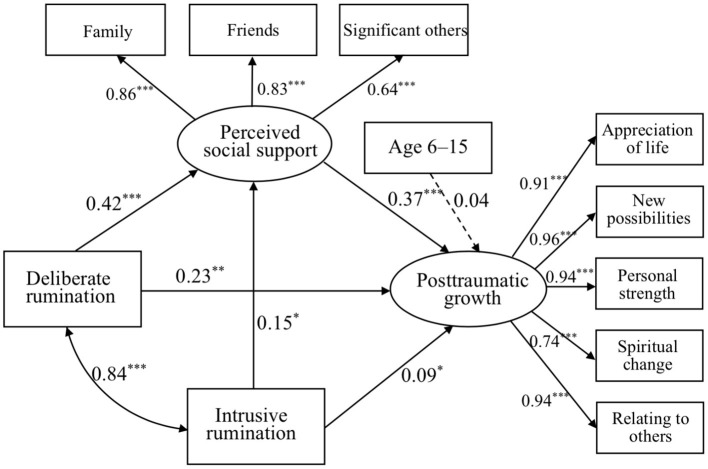
Mediation model of deliberate and intrusive ruminations to PSS and to PTG in age 6–15 sample. *p < 0.05, **p < 0.01, and ***p < 0.001.

**Figure 3:**
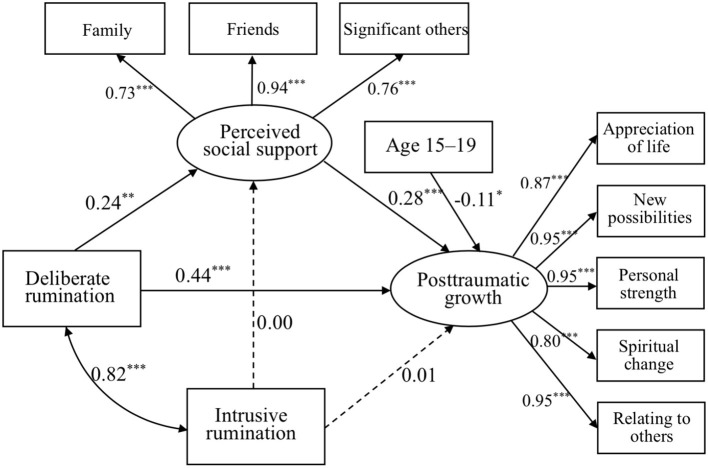
Mediation model of deliberate and intrusive ruminations to PSS and to PTG in age 15–19 sample. *p < 0.05, **p < 0.01, and ***p < 0.001.

Age was added to mediation model 1 to explore its main effect. Results indicated no significant effect on PTG in the full sample, while age had a positive but non-significant effect on PTG: ages 6–15 → PTG (*B* = 0.04, *p* = 0.47; mediation model 2a, see Figure 2); but a marginally negative effect on PTG: ages 15–19 → PTG (*B* = −0.11, *p* = 0.08; mediation model 2b, see Figure 3). Both models 2a and 2b showed an acceptable fit for the data, presented in Table 3.

### Moderation Analysis of PTSD Symptoms

Additionally, we examined the moderation models as proposed in hypotheses 4 and 5 using PROCESS to see if the effect of intrusive rumination or deliberate rumination and PSS on PTSD symptoms had interactions, as shown in Table 4. In moderation model 1 (full sample), the main effect of intrusive rumination on PTSD symptoms was significant (*t* = 8.53, *p* < 0.001).

**Table 4:** Linear regression analysis: main and moderate factors associated with PTSD symptoms.

	**All participants (476)**			**Age 6–15 (310)**			**Age 15–19 (187)**		
	**Estimate**	***T***	***P***	**Estimate**	***t***	***p***	**Estimate**	***t***	***p***
Intrusive rumination	1.61	4.73	<0.001	1.19	2.75	0.006	1.41	2.56	0.01
Deliberate rumination	0.23	1.27	0.21	0.27	1.23	0.22	0.40	1.41	0.16
PSSSO	−0.55	−2.28	0.02	−0.77	−2.47	0.01	−0.91	−2.41	0.02
Intrusive × Deliberate rumination	−0.02	−2.37	0.02	−0.02	−2.03	0.04	−0.03	−2.46	0.01
Intrusive rumination × PSSSO	0.28	2.00	0.05	0.04	2.40	0.02	0.06	2.53	0.01

**Figure 4:**
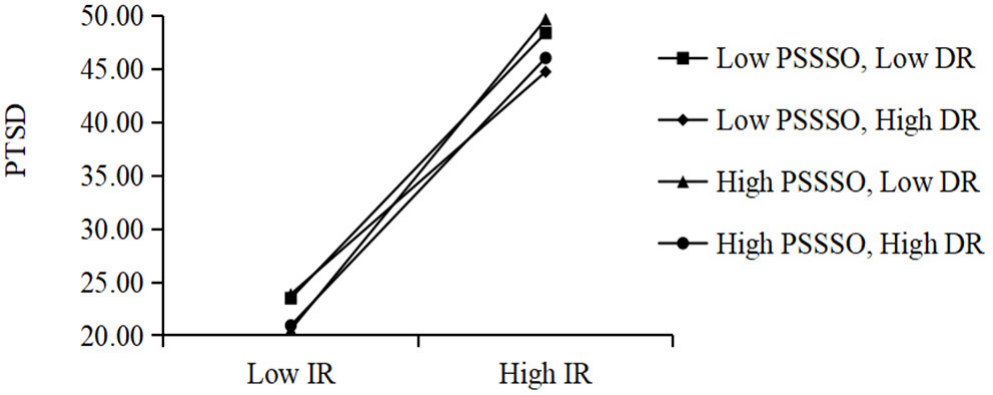
Moderation model of deliberate rumination (DR) and intrusive rumination (IR) and perceived social support from significant others (PSSSO) to PTSD symptoms.

PSSSO and its interactions with intrusive rumination were added to moderation model 1, as shown in Figure 4. The main effect of PSSSO was significant (*t* = −2.28, *p* < 0.05), while both interactions of intrusive × deliberate rumination (*t* = −2.37, *p* < 0.05) and intrusive rumination × PSSSO (*t* = 2.00, *p* = 0.05) were significant on PTSD symptoms.

A moderation model was also performed to explore subgroups of age. Similar to the full sample of moderation model 1, the main effect of intrusive rumination and PSSSO, and the interaction of intrusive × deliberate rumination and intrusive rumination × PSSSO, was significant for both age 6–15 (moderation model 2) and age 15–19 (moderation model 3) samples.

## Discussion

This study was to clarify the relationship of rumination and PTG, studying the long-term (12 years after) effect on PTG and PTSD symptoms regarding an earthquake trauma during childhood and adolescence in young survivors, and explore the mediating and moderating role of PSS in the relationship between rumination and PTG and PTSD symptoms. To our knowledge, this was the first study to examine the role of rumination and PSS in PTG and PTSD symptoms among adult Wenchuan earthquake survivors, focusing on samples of people who were children and adolescents at the time of the event.

This study showed substantial long-term consequences of traumas that occurred during childhood and adolescence, affecting PTG and PTSD in young adulthood. Remarkably, the present study found that PTG level differed significantly among participants at different age stages when the earthquake occurred. As age increased, PTG scores nearly increased from ages 6–15, and then decreased from ages 15 to 19. In addition, the results showed that PTSD symptom level also significant differed among different age stages, specifically, survivors aged 12–15 reported the highest level of PTSD symptoms. Results indicated that long-term effects of PTG and PTSD symptoms would significantly differ among different non-adult age groups who suffered the same earthquake. As age increased, PTG scores increased from ages 6–15, while they decreased from ages 15–19. A possible explanation for the difference is that survivors in early adolescence who experienced the earthquake were in a stage of rapid physical, psychological, and brain development when cognition and emotion are susceptible to trauma impact, compared with survivors in childhood and late adolescence (7, 40). This finding complements current views of child development and highlights that the drastic transition period from childhood to adolescence is marked by age-related changes in cognitive, emotional, and social processes that may influence the likelihood of psychological outcomes following trauma exposure beyond the role of sociodemographic factors (7). Trauma exposure in adolescence should be of particular concern and targeted in psychological prevention and protection.

Deliberate rumination was found to be most positively associated with PTG, followed by intrusive rumination. For adolescent survivors during the Wenchuan earthquake, PTG was affected by many factors, especially related to rumination and PSS for youth survivors. As a form of vulnerable cognition, rumination began in the aftermath of the earthquake, and it challenged the framework by which the children understood the world. Rumination, both negative, distressing thoughts, and deliberate, repetitive thinking, may play a key role in PTG (20). Additionally, intrusive rumination was most significantly associated with PTSD symptoms—this finding was consistent with previous studies (20, 30), followed by deliberate rumination. Our study also supported the idea that cognitive models of PTSD, such as ruminative cognitive styles, compelled individuals to recall negative traumatic memories and perceptions, but ultimately interfered with the process of psychological distress recovery (29, 32).

In line with prior cross-sectional and longitudinal work (1, 15, 21), despite the common co-occurrence between PTG and PTSD after traumatic exposure, this finding supported the notion that PTG and PTSD symptoms are two independent variables (4, 16), but it did not parallel other studies (2, 5, 8). A potential explanation might be attributed to variance in PTG and PTSD symptom measurement instruments and that participants and traumatic experiences varied [for example, war and earthquake experience; (8, 15)]. Given the inconclusive associations between PTG and PTSD symptoms, more research is needed to examine different traumatic experiences.

### Rumination and PTG: PSS as a Mediator

Using the full sample, as well as both the age 6–15 and 15–19 subgroups separately, our study supported the view that PSS is a protective factor for PTG (11, 55). PSS provides trauma-exposure survivors a safe environment where they can talk freely with others about traumatic experiences and associated perceptions and emotions, and reframe traumatic events and reconstruct their worldviews, thus promoting their PTG (24). These findings were in accordance with the main-effect hypothesis of social support (4) and Tedeschi and Calhoun's model of PTG (24).

Most importantly, these findings indicated that PSS mediated the relationship between intrusive rumination, deliberate rumination, and PTG for earthquake youth survivors, providing empirical evidence for the long-term effects of traumatic rumination on adulthood PTG related to PSS. Mediation model 1 revealed that intrusive rumination has an indirect effect on PTG via PSS. Deliberate rumination also has an indirect effect on PTG *via* PSS. This finding confirmed the notion that deliberate rumination could help individuals seek and receive more PSS to understand traumatic events, while PSS can lessen the negative impact of rumination on individuals and increase positive experiences, leading to PTG (19). Young adult survivors' age during the earthquake was added to mediation model 1 and indicated no significant effect on PTG among the full sample, while age had a positive effect on PTG but did not reach significance: age 6–15 → PTG (mediation model 2a); but had a marginally negative effect on PTG: age 15–19 → PTG (mediation model 2b). Findings of the mediation results confirmed hypothesis 3 that PSS would mediate the link between rumination and PTG, and the mediation model varied by age group. This finding is important because it demonstrated children and adolescents' age need to be taken into account especially for trauma-exposed PTG in the field of child development.

### Rumination and PTSD Symptoms: PSS as Moderator

In the moderation model (full sample, as well as both age 6–15 and 15–19 subgroups), the main effect of intrusive rumination on PTSD symptoms was significant and the interaction of intrusive × deliberate rumination was significant on PTSD symptoms. This finding was consistent with a previous study that suggested that intrusive rumination (but not deliberate rumination) prospectively predicts PTSD symptoms (30). The negative focus on trauma may initially invade one's cognitive world, leading one to ruminate to avoid traumatic memories and emotions, which temporarily alleviates psychological distress, but ultimately interferes with the process of adaptive recovery (29, 31). Interestingly, the findings suggest a distinct variation in intrusive rumination functioning between different levels of deliberate rumination, highlighting the critical role of the interactions in two distinct rumination dimensions for the effect on survivors' PTSD symptoms. Building upon prior rumination to PTSD research, we believe this study expands the literature by providing evidence for the significant rumination interaction mechanisms on trauma-exposed PTSD symptoms.

Additionally, in the moderation model (full sample, as well as both age 6–15 and 15–19 subgroups), the main effect of PSSSO and interaction of intrusive rumination × PSSSO were significant on PTSD symptoms. It demonstrated a variation in intrusive rumination functioning on PTSD symptoms regarding different PSSSO levels. This interaction was consistent in all three age group samples. First, we did not find family or friends' PSS as main effects or moderators on PTSD symptoms besides PSSSO. Cultural/regional diversity may account for associations between different resources of PSS and PTSD symptoms (56, 57). For example, in the long-term effects following a Turkish earthquake, neither PSS from friends nor from teachers was significantly associated with PTSD symptoms among adolescent survivors (58). After the Wenchuan earthquake, support from significant others—the Chinese government, soldiers, volunteers, psychologists, and social enterprises—provided necessary resources for children and adolescents surviving the earthquake to cope with loss and distress, especially in their house reconstruction, medical services, and psychological recovery (56, 59). Therefore, PSSSO can buffer the effects of traumatic experiences on PTSD symptoms. In addition, survivors with frequent intrusive rumination may more frequently express their dissatisfaction with their PSSSO networks and may engage in co-rumination during social interactions, which will in turn precipitate or exacerbate PTSD symptoms (47). Although rumination increases attention on negative aspects of the trauma experience, the derived negative emotions from these thoughts should be ameliorated by others' understanding and help provided in a supportive environment, and thus PTSD symptoms will be also ease (35). Therefore, PSSSO can buffer the effects of traumatic experiences and cognitions on PTSD symptoms (10). This finding confirmed the social-cognitive theory (60) that PSS was crucial in perpetuating or breaking the pathway from trauma experiences to PTSD via rumination. Our findings provide evidence that PSSSO is a preventive factor for the development of PTSD (10) and has implications for practical PTSD symptom interventions for earthquake survivors, by taking into account PSS especially PSSSO.

### Clinical Implications

It is crucial to enhance clinical outcomes for those who are considered at high risk for trauma-related psychopathology such as PTSD symptoms. Our data suggest that one potential way to prevent the onset or mitigate the extent of PTSD symptom is to provide clients with substantial social support. The availability of social support can facilitate a process of change by eliciting communication about traumatic experiences (23, 24) and lead individuals to reframe these and reconstruct their worldviews after trauma, thus potentiating PTG and mitigating PTSD symptoms.

Some individuals may ruminate to avoid traumatic memories and associated negative emotions, which temporarily alleviates distress, but ultimately interferes with the process of adaptive recovery. Therefore, counseling psychologists need to take notice of this cognitive process in clients. While helping their clients to cope with PTSD symptoms, counseling psychologists should be aware of the possibility of positive change after the trauma. In doing so, counseling interventions might focus on techniques that help clients to view PTSD symptoms and their cumulative impact with greater self-directed compassion. On that basis, counseling services may provide an opportunity to offset trauma-disclosure inhibitions that may hinder the experience of situational adaptation in the aftermath of traumatic events.

Remarkably, our findings suggest that PTG levels significantly differed in different age stages. Specifically, older adolescents reported higher levels of PTG. This may be of particular importance for emergency service professionals, especially for those who deal with the problems of children and adolescents.

### Limitations

This study employed a mono-method of gathering data by using questionnaires that could be impacted by self-report issues such as social desirability biases. As this study was cross-sectional, the causality and time-dependent fluctuations between rumination, PSS, PTG, and PTSD symptoms could not be proven. It was also noted that PTSD symptoms may have been caused by shared factors not examined in this study. Third, it is unknown whether the effects of trauma exposure persisted continuously since the event, increased over time, reappeared after periods of remission, or followed other non-linear trajectories. Prospective, longitudinal studies of trauma are needed to examine longitudinal changes in the course of posttraumatic outcomes. Except for gender and age, this study did not consider socio-demographic characteristics.

## Conclusion

Children and adolescents who experienced the Wenchuan earthquake reported a high level of PTSD symptoms as well as PTG. This study contributes new knowledge through a developmental perspective to previous theoretical and empirical studies of the relationship between rumination, PSS, and PTG/PTSD symptoms, and further suggests that PSS plays a mediating role between rumination and PTG and a moderating role between rumination and PTSD symptoms. From intervention and health-enhancement perspectives, this study highlights important implications for child and adolescent survivors of the Wenchuan earthquake.

## Data Availability Statement

The raw data supporting the conclusions of this article will be made available by the authors, without undue reservation.

## Ethics Statement

The studies involving human participants were reviewed and approved by the Ethics Committee of Sichuan University. The patients/participants provided their written informed consent to participate in this study.

## Author Contributions

WX and CF contributed to conception and design of the study, organized the database, performed the statistical analysis, and wrote the first draft of the manuscript. WX managed data collection. WX, CF, WT, and YY contributed to manuscript revision. YY contributed to potential funding support. All authors contributed to the article and approved the submitted version.

## Funding

This research was supported by the National Social Science Foundation Project (18BZZ044), Sichuan Province Science and Technology Project (20RKX0748), and Sichuan Province Major Social Science Project (SC21EZD051).

## Conflict of Interest

The authors declare that the research was conducted in the absence of any commercial or financial relationships that could be construed as a potential conflict of interest.

## Publisher's Note

All claims expressed in this article are solely those of the authors and do not necessarily represent those of their affiliated organizations, or those of the publisher, the editors and the reviewers. Any product that may be evaluated in this article, or claim that may be made by its manufacturer, is not guaranteed or endorsed by the publisher.

## References

[1] Wang W Wu X Lan X. Rumination mediates the relationships of fear and guilt to posttraumatic stress disorder and posttraumatic growth among adolescents after the Ya'an earthquake. Eur J Psychotraumatol. (2020) 10:1704993. 10.1080/20008198.2019.1704993 32002139 PMC6968513

[2] Frazier P Conlon A Glaser T. Positive and negative life changes following sexual assault. J Consult Clin Psych. (2001) 69:1048–55. 10.1037/0022-006X.69.6.1048 11777108

[3] Widows MR Jacobsen PB Booth-Jones M Fields KK. Predictors of posttraumatic growth following bone marrow transplantation for cancer. Health Psychol. (2005) 24:266–73. 10.1037/0278-6133.24.3.266 15898862

[4] Lee D Yu ES Kim NH. Resilience as a mediator in the relationship between posttraumatic stress and posttraumatic growth among adult accident or crime victims: the moderated mediating effect of childhood trauma. Eur J Psychotraumatol. (2020) 11:1704563. 10.1080/20008198.2019.1704563 32002138 PMC6968590

[5] Sleijpen M Haagen J Mooren T Kleber RJ. Growing from experience: an exploratory study of posttraumatic growth in adolescent refugees. Eur J Psychotraumatol. (2016) 7:28698. 10.3402/ejpt.v7.28698 26886487 PMC4756627

[6] Karatzias T Chouliara Z Power K Brown K Begum M McGoldrick T. Life satisfaction in people with post-traumatic stress disorder. J Ment Health. (2013) 22:501–8. 10.3109/09638237.2013.819418 24205829

[7] Ogle CM Rubin DC Siegler IC. The impact of the developmental timing of trauma exposure on PTSD symptoms and psychosocial functioning among older adults. Dev Psychol. (2013) 49:2191–200. 10.1037/a0031985 23458662 PMC3806884

[8] Solomon Z Dekel R. Posttraumatic stress disorder and posttraumatic growth among Israeli ex-pows. J Trauma Stress. (2007) 20:303–12. 10.1002/jts.20216 17597131

[9] Solomon Z Mikulincer M. Trajectories of PTSD: A 20-year longitudinal study. Am J Psychiatry. (2006) 163:659–66. 10.1176/ajp.2006.163.4.659 16585441

[10] Kakaje A Zohbi RA Aldeen OH Makki L Alyousbashi1 A Alhaffar MA. Mental disorder and PTSD in Syria during wartime: a nationwide crisis. BMC Psychiatry. (2021) 21:2. 10.1186/s12888-020-03002-3 33388026 PMC7778805

[11] Jia X Ying L Zhou X Wu X Lin C. The effects of extraversion, social support on the posttraumatic stress disorder and posttraumatic growth of adolescent survivors of the Wenchuan earthquake. PLoS ONE. (2015) 10:e0121480. 10.1371/journal.pone.0121480 25815720 PMC4376870

[12] Hamam AA Milo S Mor I Shaked E Eliav AS Lahav Y. Peritraumatic reactions during the COVID-19 pandemic-The contribution of posttraumatic growth attributed to prior trauma. J Psychiatr Res. (2021) 132:23–31. 10.1016/j.jpsychires.2020.09.029 33038562 PMC7525333

[13] Stewart AE. Trauma and transformation: growing in the aftermath of suffering. J Constr Psychol. (1998) 11:333–339.

[14] Purgato M Gastaldon C Papola D van Ommeren M Barbui C Tol WA. Psychological therapies for the treatment of mental disorders in low- and middle-income countries affected by humanitarian crises. Cochrane Database Syst Rev. (2018) 7:CD011849. 10.1002/14651858.CD011849.pub2 29975811 PMC6513488

[15] Wu X Zhou X Wu Y An Y. The role of rumination in posttraumatic stress disorder and posttraumatic growth among adolescents after the Wenchuan earthquake. Front Psychol. (2015) 6:1335. 10.3389/fpsyg.2015.01335 26388826 PMC4559646

[16] Wang W Wu X Tian Y. Mediating roles of gratitude and social support in the relation between survivor guilt and posttraumatic stress disorder, posttraumatic growth among adolescents after the Ya'an Earthquake. Front Psychol. (2018) 9:2131. 10.3389/fpsyg.2018.02131 30455660 PMC6230928

[17] Vloet TD Vloet A Bürger A Romanos M. Post-traumatic growth in children and adolescents. J Trauma Stress Dis Treat. (2017) 6:4. 10.4172/2324-8947.1000178

[18] Choi S Lemberger-Truelove ME Cho SM. The influence of disruption of core beliefs, social support, and rumination on posttraumatic growth for Korean undergraduate students. J Humanist Couns. (2019) 58:223–32. 10.1002/johc.1212125855820

[19] Xu W Jiang HL Zhou YY Zhou LL Fu H. Intrusive rumination, deliberate rumination, and posttraumatic growth among adolescents after a tornado. J Nerv Ment Dis. (2019) 207:152–6. 10.1097/NMD.0000000000000926 30807514

[20] Kilmer RP Gil RV. Exploring posttraumatic growth in children impacted by Hurricane Katrina: Correlates of the phenomenon and developmental considerations. Child Dev. (2010) 81:1211–27. 10.1111/j.1467-8624.2010.01463.x 20636691 PMC2907541

[21] Zhou X Wu X. The relationship between rumination, posttraumatic stress disorder, and posttraumatic growth among Chinese adolescents after earthquake: a longitudinal study. J Affect Disord. (2016) 193:242–8. 10.1016/j.jad.2015.12.076 26773915

[22] Kilmer RP Gil-Rivas V Griese B Hardy SJ Hafstad GS Alisic E. Posttraumatic growth in children and youth: Clinical implications of an emerging research literature. Am J Orthopsychiatry. (2014) 84:506–18. 10.1037/ort0000016 25110973

[23] Sutton V Robbins I Senior V Gordon S. A qualitative study exploring refugee minors' personal accounts of post-traumatic growth and positive change processes in adapting to life in the UK. Divers Health Soc Care. (2006) 3:77–88.

[24] Tedeschi RG Calhoun LG. Posttraumatic growth: conceptual foundations and empirical evidence. Psychol Inq. (2004) 15:1–18. 10.1207/s15327965pli1501_01

[25] Cohen S Wills TA. Stress, social support, and the buffering hypothesis. Psychol Bull. (1985) 98:310–57. 10.1037/0033-2909.98.2.310 3901065

[26] Macksoud MS Aber JL. The war experiences and psychosocial development of children in Lebanon. Child Dev. (1996) 67:70–88. 10.1111/j.1467-8624.1996.tb01720.x 8605835

[27] Taku K Cann A Tedeschi RG Calhoun LG. Intrusive versus deliberate rumination in posttraumatic growth across US and Japanese samples. Anxiety Stress Coping. (2009) 22:129–36. 10.1080/10615800802317841 18937084

[28] Moulds ML Bisby MA Wild J Bryant RA. Rumination in posttraumatic stress disorder: a systematic review. Clin Psychol Rev. (2020) 82:101910. 10.1016/j.cpr.2020.101910 32971312

[29] Papageorgiou C Wells A. An empirical test of a clinical metacognitive model of rumination and depression. Cogn Ther Res. (2003) 27:261–73. 10.1023/A:1023962332399

[30] Jenness JL Jager-Hyman S Heleniak C Beck AT Sheridan MA McLaughlin KA. Catastrophizing, rumination, and reappraisal prospectively predict adolescent PTSD symptom onset following a terrorist attack. Depress Anxiety. (2016) 33:1039–47. 10.1002/da.22548 27557454 PMC5325818

[31] Ehlers A Clark DM. A cognitive model of posttraumatic stress disorder. Behav Res Ther. (2000) 38:319–45. 10.1016/S0005-7967(99)00123-0 10761279

[32] Michael T Halligan SL Clark DM Ehlers A. Rumination in posttraumatic stress disorder. Depress Anxiety. (2007) 24:307–17. 10.1002/da.20228 17041914

[33] Lee JS. Perceived social support functions as a resilience in buffering the impact of trauma exposure on PTSD symptoms via intrusive rumination and entrapment in firefighters. PLoS ONE. (2019) 14:e0220454. 10.1371/journal.pone.0220454 31374563 PMC6677557

[34] Lepore SJ. A social–cognitive processing model of emotional adjustment to cancer. In: Baum A Andersen BL editors. Psychosocial Interventions for Cancer. Washington DC: American Psychological Association (2001). p. 99–116. 10.1037/10402-006

[35] Nelson Goff BS Summers K Hartman K Billings A Chevalier M Hermes H. Disclosure of war deployment experiences: a qualitative study of the relationship impact on military couples. Mil Behav Health. (2015) 3:190–8. 10.1080/21635781.2015.1055865

[36] Scott SB Poulin MJ Silver RC. A lifespan perspective on terrorism: age differences in trajectories of response to 9/11. Dev Psychol. (2013) 49:986–98. 10.1037/a0028916 22709132

[37] Kobayashi I Sledjeski EM Delahanty DL. Gender and age interact to predict the development of posttraumatic stress disorder symptoms following a motor vehicle accident. Psychol Trauma. (2019) 11:328–36. 10.1037/tra0000366 29446964 PMC7006741

[38] Murata S Rezeppa T Thoma B Marengo L Krancevich K Chiyka E. The psychiatric sequelae of the COVID-19 pandemic in adolescents, adults, and health care workers. Depress Anxiety. (2020) 38:233–46. 10.1002/da.23120 33368805 PMC7902409

[39] Jaen-Varas D Mari Jde J Coutinho Eda S Andreoli SB Quintana MI de Mello MF. A cross-sectional study to compare levels of psychiatric morbidity between young people and adults exposed to violence in a large urban center. BMC Psychiatry. (2016) 16:134. 10.1186/s12888-016-0847-0 27267456 PMC4896016

[40] Kar N. Depression in youth exposed to disasters, terrorism, and political violence. Curr Psychiatry Rep. (2019) 21:73. 10.1007/s11920-019-1061-9 31270638

[41] Angold A Erkanli A Silberg J Eaves L Costello EJ. Depression scale scores in 8–17-year-olds: effects of age and gender. J Child Psychol Psychiatry. (2002) 43:1052–63. 10.1111/1469-7610.00232 12455926

[42] Cole DA Tram JM Martin JM Hoffman KB Ruiz MD Jacquez FM. Individual differences in the emergence of depressive symptoms in children and adolescents: a longitudinal investigation of parent and child reports. J Abnorm Psychol. (2002) 111:156–65. 10.1037/0021-843X.111.1.156 11866168

[43] McDermott BM Palmer LJ. Postdisaster emotional distress, depression and event-related variables: findings across child and adolescent developmental stages. Aust N Z J Psychiatry. (2002) 36:754–61. 10.1046/j.1440-1614.2002.01090.x 12406117

[44] Mesman J Koot HM. Child-reported depression and anxiety in preadolescence: I. Associations with parent- and teacher-reported problems. J Am Acad Child Adolesc Psychiatry. (2000) 39:1371–8. 10.1097/00004583-200011000-00011 11068892

[45] Pine DS Cohen E Cohen P Brook J. Adolescent depressive symptoms as predictors of adult depression: moodiness or mood disorder? Am J Psychiatr. (1999) 156:133–5. 10.1176/ajp.156.1.133 9892310

[46] Fan F Zhou Y Liu X. Sleep disturbance predicts posttraumatic stress disorder and depressive symptoms: a cohort study of Chinese adolescents. J Clin Psychiatry. (2017) 78:882–8. 10.4088/JCP.15m10206 27574834

[47] Liu JE Wang HY Wang ML Su YL Wang PL. Posttraumatic growth and psychological distress in Chinese early-stage breast cancer survivors: a longitudinal study. Psychooncology. (2014) 23:437–43. 10.1002/pon.3436 25485337

[48] Blanchard EB JonesAlexander J Buckley TC Forneris CA. Psychometric properties of the PTSD checklist (PCL). Behav Res Ther. (1996) 34:669–73. 10.1016/0005-7967(96)00033-2 8870294

[49] Zhang H Shi Y Jing P Zhan P Fang Y Wang F. Posttraumatic stress disorder symptoms in healthcare workers after the peak of the COVID-19 outbreak: a survey of a large tertiary care hospital in Wuhan. Psychiatry Res. (2020) 294:113541. 10.1016/j.psychres.2020.113541 33128999 PMC7585629

[50] Shi T Jiang C Jia S Liu Q Zhang J Qi X. Severe acute respiratory syndrome evaluation scale for post-traumatic stress disorder. Chin J Clin Rehabil. (2005) 9:44–7.

[51] Cann A Calhoun LG Tedeschi RG Triplett KN Vishnevsky T Lindstrom CM. Assessing posttraumatic cognitive processes: the event related rumination inventory. Anxiety Stress Coping. (2011) 24:137–56. 10.1080/10615806.2010.529901 21082446

[52] Zhou X Wu X An Y Chen J. The roles of rumination and social support in the associations between core belief challenge and post-traumatic growth among adolescent survivors after the Wenchuan Earthquake. Acta Psychol Sin. (2014) 46:1509–20. 10.3724/SP.J.1041.2014.01509

[53] Zimet GD Dahlem NW Zimet SG Farley GK. The multidimensional scale of perceived social support. J Pers Assess. (1988) 52:30–41. 10.1207/s15327752jpa5201_2 2280326

[54] Huang L Jiang Q Ren W. The correlation study of coping style, social support and psychosomatic symptoms of cancer patients. Chin Ment Health J. (1996) 10:160–1.

[55] Shand LK Cowlishaw S Brooker JE Burney S Ricciardelli LA. Correlates of post-traumatic stress symptoms and growth in cancer patients: a systematic review and meta-analysis. Psychooncology. (2015) 24:624–34. 10.1002/pon.3719 25393527

[56] Xu KB Yuan P. Effects of three sources of social support on survivors' posttraumatic stress after the Wenchuan Earthquake. J Loss Trauma. (2014) 19:229–43. 10.1080/15325024.2013.791516

[57] Mesidor JK Sly KF. Religious coping, general coping strategies, perceived social support, PTSD symptoms, resilience, and posttraumatic growth among survivors of the 2010 earthquake in Haiti. Ment Health Relig Cult. (2019) 22:130–43. 10.1080/13674676.2019.1580254

[58] Eray S Ucar HN Murat D. The effects of relocation and social support on long-term outcomes of adolescents following a major earthquake: a controlled study from Turkey. Int J Disaster Risk Reduct. (2017) 24:46–51. 10.1016/j.ijdrr.2017.05.026

[59] Lu BQ Zeng WQ Li ZY Wen J. Risk factors of post-traumatic stress disorder 10 years after Wenchuan earthquake: a population-based case-control study. Epidemiol Psychiatr Sci. (2021) 30:e25. 10.1017/S2045796021000123 33729117 PMC8061289

[60] Vélez CE Krause ED McKinnon A Brunwasser SM Freres DR Abenavoli RM. Social support seeking and early adolescent depression and anxiety symptoms: the moderating role of rumination. J Early Adolesc. (2015) 35:1118–43. 10.1177/0272431615594460 28458442 PMC5407371

